# *In Vitro* Bioactivities of Food Grade Extracts from Yarrow (*Achillea millefolium* L.) and Stinging Nettle (*Urtica dioica* L.) Leaves

**DOI:** 10.1007/s11130-022-01020-y

**Published:** 2022-11-12

**Authors:** Enni Mannila, Francisco J. Marti-Quijal, Marta Selma-Royo, Marta Calatayud, Irene Falcó, Beatriz de la Fuente, Francisco J. Barba, Maria Carmen Collado, Kaisa M. Linderborg

**Affiliations:** 1 Food Sciences, Department of Life Technologies, University of Turku, Turku, Finland; 2 Nutrition and Food Science Area, Preventive Medicine and Public Health, Food Science, Toxicology and Forensic Medicine Department, Faculty of Pharmacy, Universitat de València, Avda. Vicent Andrés Estellés, s/n, 46100 Burjassot, València Spain; 3 Institute of Agrochemistry and Food Technology-National Research Council (IATA-CSIC), Agustin Escardino 7, 46980 Paterna, Valencia Spain

**Keywords:** Extraction optimization, Bacterial growth, Anti-inflammatory activation, Gastric health, Immunomodulation

## Abstract

Yarrow (*Achillea millefolium* L., AM) and nettle (*Urtica dioica* L., UD) are bioactive plants used commercially in functional food and supplement applications and traditionally to alleviate gastric disorders. In this work, the effects of food-grade optimized extracts of Finnish early-season AM and UD were tested on bacterial growth including potential beneficial and foodborne pathogens, as well as murine norovirus (MNV). The anti-inflammatory properties of the extracts were also tested *in vitro* by NF-κB reporter cells. The food-grade extraction was optimized with the response surface modelling in terms of total carotenoid, chlorophyll, and phenolic compounds contents and antioxidant capacities. The optimal food-grade extraction parameters were a 1-h extraction in 70% ethanol at 45 °C for AM, and at 49 °C for UD. There were no significant effects on the beneficial bacteria (*Lacticaseibacillus *and* Bifidobacterium* strains), and the extracts were more effective against gram-positive than gram-negative foodborne bacteria and potential pathogens. *Listeria innocua* was the most susceptible strain in the optimized extracts with a growth rate of 0.059 ± 0.004 for AM and 0.067 ± 0.006 for UD, *p* < 0.05 compared to control. The optimized extracts showed a logarithmic growth reduction of 0.67 compared to MNV. The hydroethanolic extracts were cytotoxic to both cell lines, whereas aqueous AM and UD extracts induced and reduced TLR4 signalling in a reporter cell line, respectively. The results provide novel food-grade extraction parameters and support the bioactive effects of AM and UD in functional food applications, but more research is needed to elucidate the precise biological activity *in vivo* for gastric health.

**Supplementary Information:**

The online version contains supplementary material available at 10.1007/s11130-022-01020-y.

## Introduction

Yarrow (*Achillea millefolium* L., AM) and stinging nettle (*Urtica dioica* L., UD) are highly bioactive traditional medicinal plants used to alleviate various symptoms, including gastric disorders [1–4]. Nowadays, their overground parts are approved for food supplement (AM) and food (UD) use in the European Union [5]. Bioactive plant extracts have multiple uses in the food and pharmaceutical industries, including functional foods or supplements, extending the shelf-life of foods, and contributing to the chemical and microbial safety of products at low cost and good consumer acceptance [6], even when proper hygiene and other safety actions cannot be completely replaced by bioactive plant compounds. Furthermore, the consumption of bioactive compounds in extracts can modulate the gut microbiota by affecting the metabolic activity and/or composition of the microbiota with effects in host physiology and health [1, 6]. This modulation could regulate inflammatory related disorders, [6] that can be induced by bacterial lipopolysaccharide (LPS). The LPS is a cell wall structure typical of gram-negative bacteria and is a triggering ligand for the immune system via the Toll-Like Receptor 4 (TLR4), which ultimately leads to the activation of the transcription factor nuclear factor kappa B (NF-κB) and the production of pro-inflammatory cytokines [7]. The anti-inflammatory properties of AM and UD are mainly associated with their phenolic compounds [1, 3, 8].

The chemical compositions of AM and UD have been previously determined, with both plants containing different phenolic and other bioactive compounds [9, 10]. In addition to anti-inflammatory effect, AM has demonstrated many bioactive properties, such as antioxidant, antimicrobial and antiproliferative [3, 11, 12] as well as modulating effects on the intestinal microbiota [1]. As for UD, it has shown antioxidant, antimicrobial and antitumour properties [4, 9, 12], and has also been shown to be a good source of bioactive chlorophylls and carotenoids, especially lutein and *β*-carotene [4, 13]. The growth location and harvest time of plants contribute to their composition of bioactive compounds [9, 14, 15], yet the properties of AM and UD collected for food in the early season from northern latitudes, such as Finland, have not been studied so far.

The properties of the plant extract are influenced not only by the type, place of growth and season, but also by the extraction process. Extraction should be optimized to yield maximal concentrations of polyphenols and other bioactive compounds. The response surface methodology (RSM) consists of mathematical and statistical methods that can correlate the relationships between the interactions of independent variables and responses and find the combinations of them that give the optimal parameters for the extraction [16]. RSM has previously been used to optimize the extraction of antioxidants from AM waste dust [17] and the extraction of phenolic compounds from UD leaves [18].

Thus, the aim of this study was to optimize the food-grade extraction of AM and UD collected early in the Finnish growing season (May) and unravel potential intestinal health related bioactive effects *in vitro* to facilitate future studies on *in vivo* models. Extracts optimized with RSM were hypothesized to contain more bioactive compounds than aqueous extracts and thus to show greater bioactivity. The profiles of the extracts were assessed, and the antioxidant, antiviral, and anti-inflammatory activities of the extracts as well as their effect on potential pathogens and beneficial bacterial growth were evaluated.

## Materials and Methods

A detailed description of the materials and methods can be found in the supplementary material (ESM 1).

## Results and Discussion

### Optimization of the Extraction

The optimization results and quadratic functions are fully reported in the supplementary material (ESM 2). To summarize, the optimization with RSM was considered successful. The extraction parameters significantly influenced the compositions of the extracts, and the optimal conditions for extraction were found with RSM when 45 °C, 70% ethanol (EtOH) and 1 h were used for AM and 49 °C, 70% EtOH and 1 h for UD. The extraction of AM and UD has been optimized using different methods, and here, the optimal time (1 h) for AM is shorter and for UD longer than in the previous literature [17–19]. The temperatures of 45 and 49 °C obtained here are reasonable as a temperature of 40 °C has been reported as good for obtaining spinach extracts high in carotenoids [20]. An EtOH concentration of 70% is rather high, but consistent with a previous study [17]. The plants in the present study are from the early season as young leaves are preferred for food use and early season UD contains the highest levels of bioactive compounds [4, 9]. Our results are similar to previous AM and UD extraction results [11, 17–19]. The measured total carotenoids, total chlorophylls, total phenolic compounds, and antioxidant capacities are represented for each parameter setting of the Box-Behnken design in ESM 2 Tables S1–S2.

### Effects on Bacterial Kinetics

**Fig. 1:**
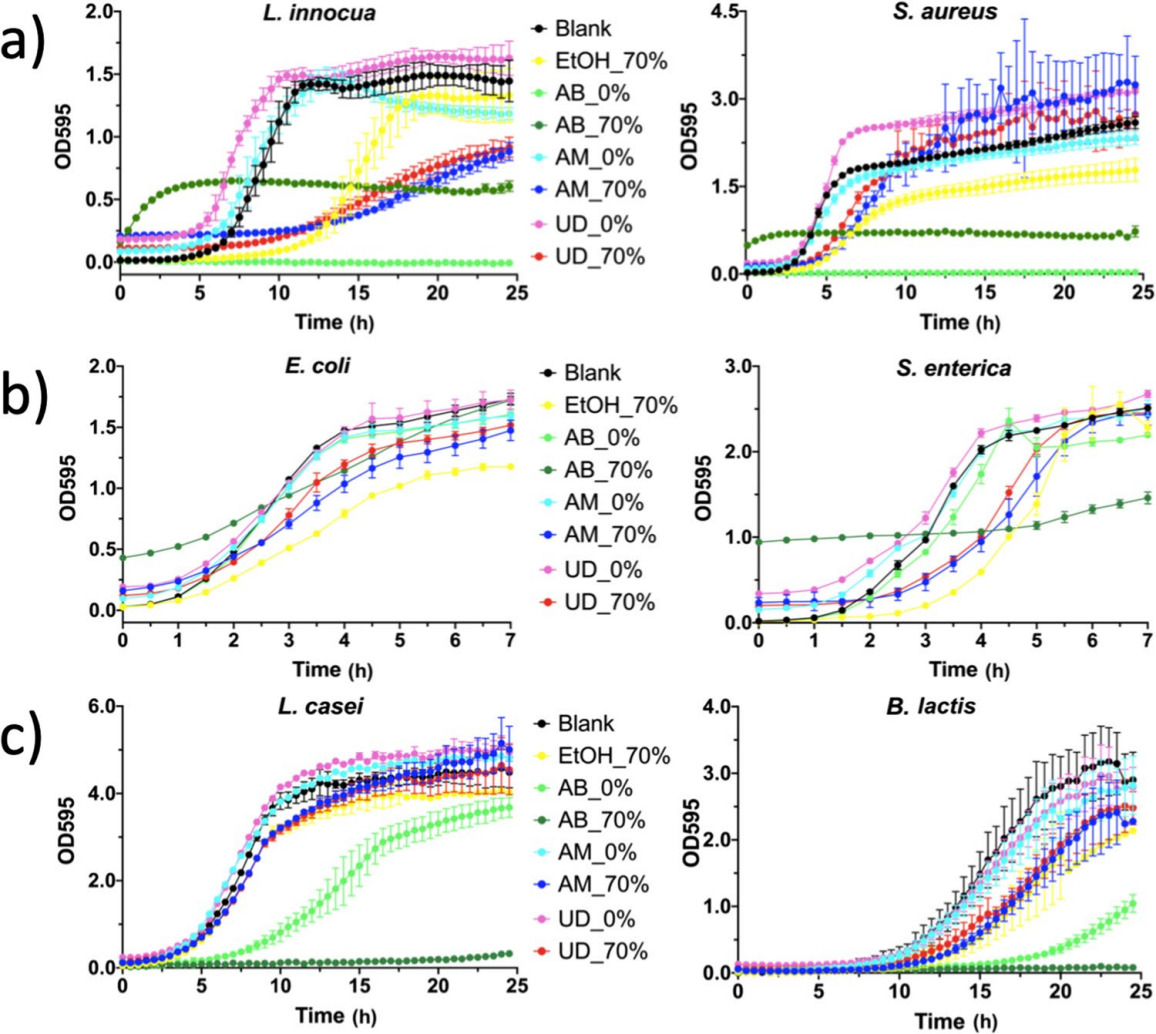
Growth curves of **a**) gram-positive *L. innocua* and *S. aureus*, **b**) gram-negative *E. coli* and *S. enterica*, and **c**) *L. casei* and *B. lactis*. OD: Optical density, Blank: culture medium, EtOH_70%: 70% ethanol, AB: antimicrobial blend in water (0%) and in 70% ethanol, AM: *A. millefolium* in water (0%) and in 70% ethanol, UD: *U. dioica* in water (0%) and in 70% ethanol. The datapoints are from five replicates with standard deviation

The growth curves of the bacterial strains tested were analysed considering the effects of the extracts on the maximal optical density (MOD), growth rate and lag of growth of each bacterial strain which are fully detailed in the Supplementary Material (ESM 3). In short, the bacterial strain and the solvent used influenced the growth of the bacteria (Fig. 1), as previously reported [11, 12, 21]. The gram-positive bacteria (*L. innocua* and *S. aureus*) were more susceptible to the ethanolic extracts than gram-negative (*E. coli* and *S. enterica*), and AM reduced the growth better than UD. On the contrary, UD enhanced the bacterial growth, possibly due to some compounds of UD, such as certain carotenoids or polyphenols, which may have acted as bacterial substrates. Of all the strains, *L. innocua* was the most sensitive to the optimized extracts (mean growth rate with standard deviation of AM: 0.059 ± 0.004, and that of UD: 0.067 ± 0.006, *p* ≤ 0.030, compared to controls). Still, the antimicrobial blend (positive control, AB) in 70% EtOH (AB70) and in sterile water (AB0) reduced the growth of these gram-positive bacteria the most, especially AB0, which completely inhibited growth. In gram-negative *E. coli* and *S. enterica*, growth was diauxic and only the first phase of growth was analysed. The gram-negative bacteria were not significantly affected by the extracts, possibly due to the cells’ outer membrane, which can reduce the influx of antibacterial molecules [22]. Also in previous studies, *E. coli, L. monocytogenes* and *S. enterica* sv. Typhimurium were very resistant to UD [23].

Ethanol influenced the growth rate and the MOD of *S. aureus* and *E. coli* and also, the lag of growth of *L. innocua*, *S. aureus* and *S. enterica* compared to the control media. In these cases, the ethanolic extracts were not compared to the aqueous versions due to the EtOH effect.

Previous studies found that gram-positive bacteria are more susceptible to plant extracts than gram-negative ones [1, 17]. UD is considered to be more effective than AM on antibacterial control against both gram-positive and negative bacteria, mainly due to its total phenolic and flavonoid compound contents [12]. Since the plants in the present study are from the early growing season, the phenolic compounds may not be as abundant, or the antibacterial effects as high as in the more mature plants in the previous studies. Previously, hydroethanolic extracts were found to be more effective against bacteria than aqueous ones [21]. Nevertheless, Mahmoudi *et al.* [24] found that an aqueous extract of UD leaves was more antibacterial than the corresponding ethanolic extract, which was attributed to more efficient water extraction conditions. This highlights the importance of optimizing of the extraction according to the desired bioactivity and related compounds.

Regarding the potentially beneficial bacteria tested, they were more affected by the AB than by the extracts, indicating a neutral effect of the extracts (Fig. 1c). No significant differences were found compared to the extracts and the control blanks. *Lacticaseibacillus* strains are considered to be more resistant to the antibacterial compounds than the pathogenic gram-positive and -negative bacteria and can even use the compounds as substrates, which could explain the behaviour [25]. Kozłowska *et al.* [26] found that lactic acid bacteria were not inhibited by spice extracts, such as UD powder extracted with 70% EtOH, but Milutinović *et al.* [1] showed growth stimulation of probiotic *Saccharomyces boulardii* and *L. plantarum* with AM extract.

### Antiviral Activity

No significant differences were reported for aqueous extracts, reductions of 0.25 and 0.63 log were found for AM and UD, respectively (Table 1). For ethanolic extracts, the titers decreased in 0.67 log for both and showed statistical variations from PBS and ethanol control. No effects result from ethanol control. In our previous work, an aqueous 1/10 dilution of *E. glaucophyllum* extract reported 0.62 logarithmic reduction for murine norovirus (MNV) which is similar to the results obtained in this study for aqueous UD [21]. Still, the ethanolic extract of *E. glaucophyllum* (as 1/10 dilution of 50% EtOH) compared to optimized UD extract resulted in higher reduction with 1.92 log.

**Table 1:** Reduction of murine norovirus (MNV) titers (log TCID_50_/ml) treated with *A. millefolium* (AM) and *U. dioica* (UD) extracts overnight (o/n) at 37 °C. The aqueous (H_2_O) and ethanolic (70%) extracts were diluted 1/10 with phosphate-buffered saline (PBS)

MNV (37 °C, o/n) log TCID_50_/ml		
Treatment	Recovered titer	log Reduction
Control PBS	5.07 ± 0.35^a^	–
AM H_2_O 1/10	4.82 ± 0.22^ab^	0.25
UD H_2_O 1/10	4.45 ± 0.22^ab^	0.63
Control EtOH	4.89 ± 0.09^ab^	0.19
AM 70% 1/10	4.41 ± 0.26^b^	0.67
UD 70% 1/10	4.41 ± 0.07^b^	0.67

^TCID50/ml: Median (50%) tissue culture infectious dose^

^log Reduction: logarithmic reduction (subtracted from the control PBS)^

^Different letters denote significant differences between treatments at level 0.05^

Aqueous extracts have been observed to have a low potential against viral infection also previously [27]. Falcó *et al.* [28] tested the effect of a flavonoid epigallocatechin gallate of green tea on MNV and the reduction was 1.16 log for 1/10 dilution. Compared to these studies the antiviral activity of AM and UD extracts was existing but not as major. Natural compounds are studied as an option for their antiviral activities [29], but antiviral activity of plant extracts is still relatively poorly studied, and mostly reported for green tea extract and its catechins, such as epigallocatechin which shows potential activity against hepatitis A virus and MNV [28].

### Effects of Extracts on Cell Viability

The viability of HEK-Blue™ hTLR4 cells treated with varying concentration of EtOH was above 89% (*p* > 0.05) compared with cells exposed to cell culture media and thus, EtOH did not show any cytotoxic effects. However, EtOH was cytotoxic to the NFΚβ-HT-29 reporter cell line (cell viability 11–96%, *p* < 0.05 compared to respective control). The different origin of cell lines, from human embryonic kidney or colon adenocarcinoma could affect the sensitivity of cell lines to EtOH. Combined cytotoxicity between extracted compounds and EtOH cannot be excluded. All dilutions of AM70 and UD70 as 1/10 and 1/20 dilutions showed cytotoxicity (cell viability 12–87%, *p* < 0.05 compared to respective control) in both cell lines and therefore these cases were omitted. Previously, extracts of UD radix have shown cytotoxicity on human colon tumorigenic cell line HT-29 cells [30]. The viability results of the cells are provided fully in the supplementary material (ESM 4).

### Anti-Inflammatory Activity

**Fig. 2:**
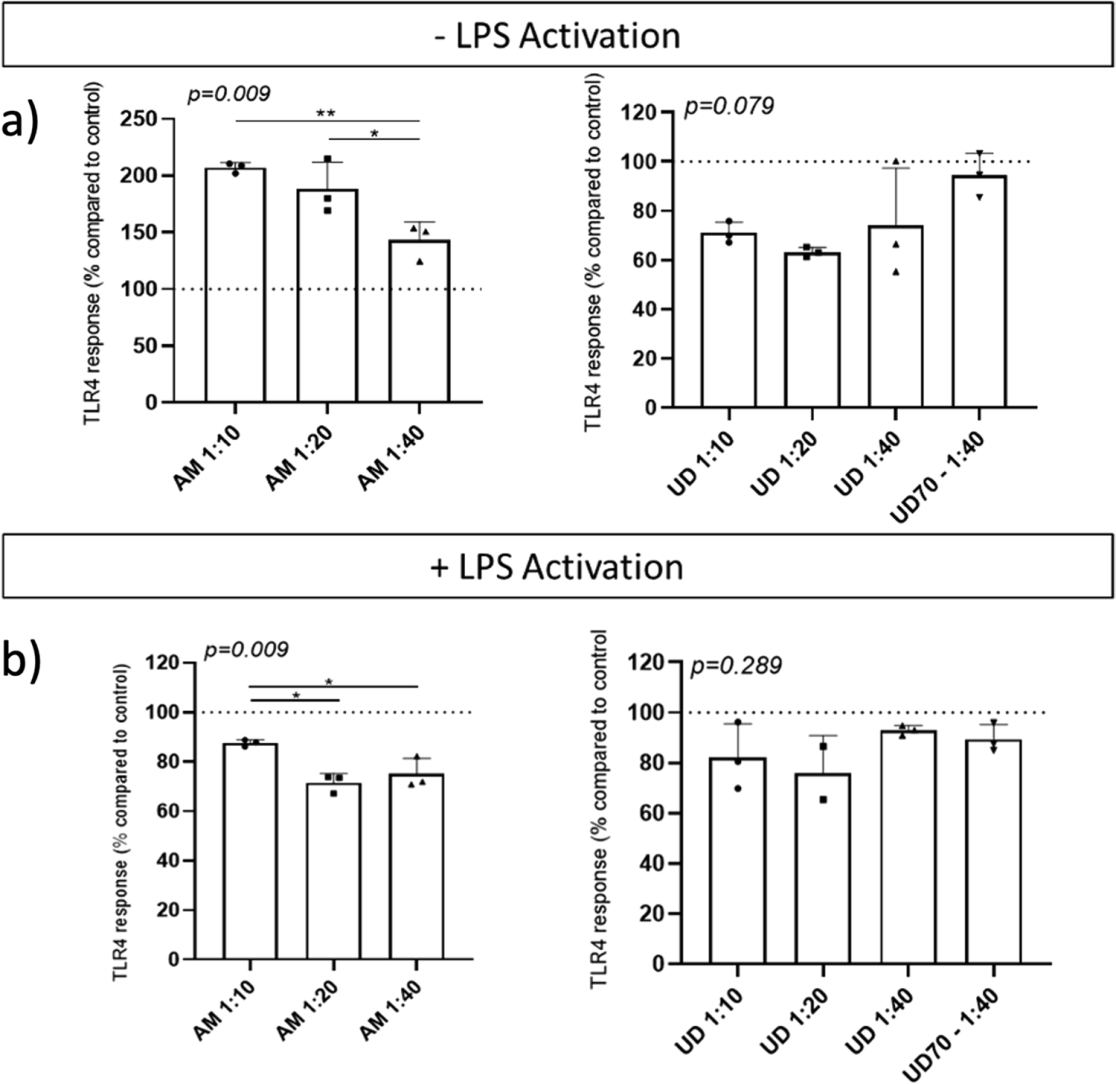
Activation of Toll-Like Receptor 4 (TLR4) in HEK-Blue™ hTLR4 cells with *A. millefolium* and *U. dioica* extracts without (**a**) and with (**b**) LPS stimulation. Data is presented as induction of TLR4 signal compared to the untreated cells signals (identified as a discontinuous line in the plots). The asterisks above the bars indicate significant differences: * *p* < 0.05, ** *p* < 0.01 between the extract dilutions. AM: aqueous extract of *A. millefolium*; UD70: *U. dioica* extract in 70% EtOH; UD: aqueous extract of *U. dioica*; LPS: Lipopolysaccharide. 1/10, 1/20 and 1/40 after the sample name refer to the used dilution

The optimized ethanolic extracts and their aqueous counterparts were tested for activation and/or inhibition of activated TLR4 pathway (Fig. 2). The hydroethanolic extracts were mostly cytotoxic to cells and thus their anti-inflammatory activity could have not been measured. Thus, no results are presented for AM70 extracts and most of the dilutions of UD70. Contrarily, aqueous extracts were not cytotoxic and in basal conditions (*i.e.*, no LPS), AM0 induced TLR4 signaling in a dose dependent manner (Fig. 2a). AM0 (1/10 dilution) was found to activate TLR4 more than AM0 (1/40 dilution) (*p* = 0.009). Similar effect of the extracts was observed in those cells stimulated with LPS (Fig. 2b), showing higher induction of TLR4 signals in those cells treated with the more concentrated samples of AM0 (*p* = 0.009). For UD, aqueous extracts (1/20 dilution) significantly diminished the TLR4 activation (Fig. 2a) compared to control (Tukey’s test C vs UD 1/20, *p* = 0.023) while UD 1/10 showed a tendency towards the same effect (Tukey’s test C vs UD 1/10, *p* = 0.070). No effect was detected in UD70 in terms of TLR4 activation compared to controls. No statistically significant differences were found among the treated cells with the LPS induction (Fig. 2b). To study the function of different extracts on human TLR4 activation, we used HEK293 cells stably co-transfected with the TLR4, MD-2 and CD14 co-receptor genes. TLR4 activation induces secreted embryonic alkaline phosphatase reporter gene, placed under the control of an IL-12 p40 minimal promoter fused to NF-κB and AP-1-binding sites. In concordance with our results, previous studies have showed that methanol extracts of UD induce the secretion of chemokines by intestinal epithelial cells independently of TLR4 activity, whereas the main signaling path was MyD88/NF-κB [31]. UD extracts had a mild reduction of TLR4 activation in non-LPS stimulated cells. UD has been previously described to show inhibition of NF-κB activation in various cell lines [32], but TLR4-independent mechanisms could be involved. Interestingly, AM extracts showed a different effect on TLR4 activation depending on the presence of LPS. Burk *et al.* [33] showed that an aqueous extract of AM inflorescences suppresses pro-inflammatory responses in LPS-induced murine macrophage cell line. We observed a similar trend in human TLR4 reporter cell line, supporting that AM bioactive molecules can modulate inflammatory responses mediating NF-κB modulation via TLR4 signalling cascade. Contrarily, AM extracts under non-inflamed conditions promoted TLR4 activation. Freysdottir *et al.* [34] isolated an AM polysaccharide (Am-25-d) with ability to induce cytokine production (*i.e*., IL-1β, IL-8, IL-10, IL-12p40, IL-23 and TNF-α) in M1 induced THP-1 macrophages, suggesting immunoenhancing properties that may be mediated via the Akt pathway. In addition, AM has previously shown inhibition of LPS-induced NO secretion and a significant effect on free radical scavenging [35]. Our results suggest that AM extracts can mediate immunomodulatory activities depending on the basal conditions, enhancing TLR4 mediated responses in non-inflamed conditions and reducing pro-inflammatory signals under pathogenic LPS presence.

## Conclusions

This study provides novel information on bioactivities and optimization of food-grade extraction of specific and traditional Finnish *A. millefolium* and *U. dioica* from an early growing season. The results show that the extracts exhibit bioactive effects *in vitro*, such as antioxidant capacity, antibacterial effects on gram-positive bacteria, a slight antiviral activity and anti-inflammatory effects. The optimization was deemed successful and the design adequate. Ethanol was found to be a more effective extraction solvent than water, as was hypothesized. This was observed with more extracted compounds and stronger antibacterial effects, especially against gram-positive bacteria. The aqueous extracts significantly or slightly improved the growth of both pathogenic and probiotic bacteria. Growth modulation of the bacteria was not as good as was hypothesized, but there were clear antibacterial effects with gram-positive bacteria, of which *L. innocua* was inhibited by the optimal extracts. However, the gram-negative bacteria were very resistant to them. AM showed more antioxidant and slightly more antibacterial effects than UD. The potential beneficial bacteria tolerated the extracts well, but they did not show as much potential for promoting the growth of these bacteria as had been assumed. More strains need to be tested in future studies. The results of *A. millefolium* and *U. dioica* for murine norovirus reduction are novel and can be confirmed in future studies with dose dependency. Hydroethanolic extracts of *A. millefolium* and *U. dioica* showed cytotoxicity towards both cell lines, with NFΚβ-HT-29 cells being the most susceptible to ethanol, and the aqueous extracts, mainly from *A. millefolium*, showed immunomodulatory activity via the TLR4 signalling cascade. As novel data for Finnish *A. millefolium* and *U. dioica*, these results give a good base of knowledge on the optimal food-grade extraction parameters. The optimization values can be used in further studies to find the best conditions for each trait or even add more parameters for more optimal extracts and for specific uses. The results support that *A. millefolium* and *U. dioica* are potential food and food supplement materials, but more research is needed to fully understand their benefits on gastric health, especially *in vivo*.

## Supplementary Information

Below is the link to the electronic supplementary material.

Supplementary file1 (PDF 310 KB)

Supplementary file2 (PDF 833 KB)

Supplementary file3 (PDF 232 KB)

Supplementary file4 (PDF 1223 KB)

## Data Availability

The data is available upon reasonable request from the corresponding authors.

## Abbreviations

AB: Antimicrobial blend (in sterile water: AB0; in 70% ethanol: AB70)
AM: *Achillea millefolium* L. (in sterile water: AM0; in 70% ethanol: AM70)
EtOH: Ethanol
LPS: Lipopolysaccharide
HEK: Human embryonic kidney cells
MNV: Murine norovirus
MOD: Maximal optical density
NF-κB: Nuclear factor kappa B
NFΚβ-HT-29: Human colon tumorigenic cells established in the Laboratory of Lactic Acid Bacteria and Probiotics of IATA-CSIC
PBS: Phosphate-buffered saline
RSM: Response surface methodology
TCID50/ml: Median (50%) tissue culture infectious dose
TLR4: Toll-like receptor 4
UD: *Urtica dioica* L. (in sterile water: UD0; in 70% ethanol: UD70)
