# Supplementary material for: In Vitro Bioactivities of Food Grade Extracts from Yarrow (Achillea millefolium L.) and Stinging Nettle (Urtica dioica L.) Leaves
Source: Plant Foods Hum Nutr. 2022 Nov 12;78(1):132–8. doi: 10.1007/s11130-022-01020-y (PMC9947014; doi:10.1007/s11130-022-01020-y)
Supplement: Supplementary file 1 — Supplementary file1 (PDF 310 KB) [file 11130_2022_1020_MOESM1_ESM.pdf]

**ESM 1**

# *In vitro* bioactivities of food grade extracts from yarrow (*Achillea millefolium* L.) and stinging nettle (*Urtica dioica* L.) leaves

Plant Foods for Human Nutrition

Enni Mannila[a] (ORCID 0000-0002-8199-8137), Francisco J. Marti-Quijal[b] (ORCID 0000-0001-9034-5325), Marta Selma Royo[c] (ORCID 0000-0002-4258-947X), Marta Calatayud[c] (ORCID 0000-0003-3592-3377), Irene Falcó[c] (ORCID 0000-0002-4036-3274), Beatriz de la Fuente[b] (ORCID 0000-0002-4157-6159), Francisco J. Barba[b] (ORCID 0000-0002-5630-3989), Maria Carmen Collado[c,*] (ORCID 0000-0002-6204-4864) & Kaisa M. Linderborg[a,*] (ORCID 0000-0003-1977-7322)

[a]Food Sciences, Department of Life Technologies, University of Turku, Turku, Finland
[b]Nutrition and Food Science Area, Preventive Medicine and Public Health, Food Science, Toxicology and Forensic Medicine Department, Faculty of Pharmacy, Universitat de València, Avda. Vicent Andrés Estellés, s/n, 46100 Burjassot, València, Spain
[c]Institute of Agrochemistry and Food Technology-National Research Council (IATA-CSIC), Agustin Escardino 7, 46980 Paterna, Valencia, Spain
[*]Corresponding authors Maria Carmen Collado (mcolam@iata.csic.es) and Kaisa M. Linderborg (kaisa.linderborg@utu.fi)

## Electronic Supplementary Material 1: Materials and methods

### Samples

Young leaves of *Achillea millefolium* L. (AM) and *Urtica dioica* L. (UD) were harvested in late May 2019 from plant specific vegetable patches at an organic farm in Hämeenkyrö, Finland (61° 37.091 North; 023° 09.168 East). Dirt and pollen were washed away with cold potable water and the water was patted away with paper towels. AM branches were spread on paper towels on tables of a food grade laboratory. UD branches were tied with jute twine as bunches of 4–5 branches and hanged on the clothes horse that was set up in a ventilated food grade fume hood. AM and UD were let dry at RT (25 ± 2 °C) avoiding direct sun light for 5 days. Then, the leaves were crushed with a hand blender (Bamix F&H of Scandinavia AS Norway; 230V~, 200W, M200; 2018-2; Switzerland) by small pulses until small pieces/powder was formed. The crushed material was then stored at 4 ± 2 °C in dark until usage.

### Extraction

A randomised sample set with 16 samples was created with Statgraphics Centurion XVI software (Statpoint Technologies, Inc., USA) and the plant material was extracted according to the obtained conditions (ESM1 Table S1) by allowing to stir at 300 rpm on a heating plate (MultiMixHeat, MMH90E, OVAN, Spain). The crude extracts were centrifuged (4 °C, 2880 x g, 15 min) in an Eppendorf Centrifuge 5810 R (Eppendorf AG, Germany). Samples were filtered under darkness to avoid photolysis and the tubes were covered with aluminium foil. The samples were stored at –24 ± 4 °C.

***ESM1 Table S1*** *Summary of randomised settings of independent variables for Box-Behnken design in A. millefolium and U. dioica extracted with a solute ratio 1/24, w/v.*

| Run | Factor $X_1$: Temperature (°C) | Factor $X_2$: Ethanol (%) | Factor $X_3$: Time (h) | Run | Factor $X_1$: Temperature (°C) | Factor $X_2$: Ethanol (%) | Factor $X_3$: Time (h) |
|---|---|---|---|---|---|---|---|
| 1 | 35 | 35 | 12.5 | 9 | 20 | 35 | 12.5 |
| 2 | 50 | 70 | 24 | 10 | 35 | 35 | 12.5 |
| 3 | 50 | 35 | 12.5 | 11 | 50 | 70 | 1 |
| 4 | 35 | 0 | 12.5 | 12 | 20 | 70 | 1 |
| 5 | 35 | 70 | 12.5 | 13 | 50 | 0 | 24 |
| 6 | 20 | 0 | 1 | 14 | 35 | 35 | 24 |
| 7 | 20 | 70 | 24 | 15 | 35 | 35 | 1 |
| 8 | 20 | 0 | 24 | 16 | 50 | 0 | 1 |

**Total contents of carotenoids (TCa) and chlorophylls (TCh)**

The determination of TCa and TCh was performed as previously in our research group [1]. The absorbances of the extracts were measured at 440, 644 and 662 nm as triplicates using a spectrophotometer (Super Aquarius Cecil CE9500, 9000 Series, Cecil Instrumentation Services Ltd., UK), and contents calculated with equations from von Wettstein [2]:

$$Carotenoids \left(\frac{mg}{L}\right): (4.695 \times Abs440.5) - 0.268\ (chl.A + chl.B) \quad (1)$$

$$Chlorophyll\ a \left(\frac{mg}{L}\right): (9.784 \times Abs662) - (0.99 \times Abs644) \quad (2)$$

$$Chlorophyll\ b \left(\frac{mg}{L}\right): (21.426 \times Abs644) - (4.65 \times Abs662) \quad (3)$$

**Total phenolic content (TPC)**

The assay was carried out according to the Folin-Ciocalteu method of Singleton and Rossi [3] updated by Barba *et al.* [1]. Gallic acid (10 mg/ml) was used as a standard (0, 0.1, 0.3, 0.5, 0.7, 1.0 mg/ml) and extract samples were diluted to 1/5. Three millilitres of 2 % $Na_2CO_3$ were added to 100 µl of Folin-Ciocalteu reagent (2 N diluted 50 % in water). Next, 100 µl of each sample were added to the previous mixture and incubated in tubes under darkness for 60 min. Finally, the absorbance was measured at 765 nm. The samples were measured as triplicates and a standard curve of gallic acid was used for calculations of gallic acid equivalents (GAE).

**Trolox equivalent antioxidant capacity (TEAC) assay**

The assay was performed following the method by Re *et al.* [4] updated by Barba *et al.* [5]. and is based on the ability of the antioxidants in the sample to reduce the ABTS radical. ABTS radical was prepared using 7 mM ABTS and 140 mM $K_2S_2O_8$. The solution was kept under darkness at RT overnight and then diluted with 96 % EtOH to a working solution as giving an absorbance of 0.7 ± 0.02 at 734 nm at temperature of 28 ± 2 °C. A standard curve was obtained with 0–500 µM of trolox

by adding into a cuvette 2 ml of working solution radical and 0.1 ml of certain standard curve sample, incubating 3 minutes under darkness and then the absorbance was measured. A standard curve of trolox was used for calculations of trolox equivalents (TE).

**Oxygen radical absorbance capacity assay**

The assay was performed on a 96-well plate using a multiplate reader PerkinElmer Victor3 1420 Multilabel counter with Wallac 1420 Manager software according to Ou, Hampsch-Woodill and Prior [6], updated by Barba *et al.* [5]. In every well of the plate, 50 µl of the diluted extract samples or trolox as a standard and 50 µl of fluorescein were added. The plate was preheated at 37 °C for 10 min and a reaction with 25 µl of 2,2-azobis(2-amidinopropane) dihydrochloride (AAPH; Sigma–Aldrich Co., USA) was started and measured every 60 seconds for 61 repeats at 37 °C. The dilutions of the samples were 1/250 and 1/500 in phosphate buffer (7.5 mM, pH 7) and the final concentration in a well was 1.18 µg/ml for fluorescein, 12 mg/ml for AAPH and 100 µM for trolox.

**Optimization with Response Surface Methodology (RSM)**

To investigate the simultaneous effects of solute concentration, temperature and time on TCa and TCh, TPC and antioxidant capacities, RSM with a Box-Behnken design was applied by using Statgraphics Centurion XVI software (Statpoint Technologies, Inc., USA). The maximum, minimum and central values of each independent variable (ethanol (EtOH) concentration between 0 and 70 %, temperature between 25 and 50 °C and time between 1 and 24 hours) were applied. As a result, randomised sample set with 16 samples, 15 different and one condition as duplicate, was created (ESM1 Table S1). Eight of the samples were treated on the first day and the last eight the following day.

The data from the TCa, TCh, TPC and antioxidant assays was used to evaluate the optimal conditions. It was predicted that TPC has the biggest effect on bioactivity of the plants and thus weighted the most [7]–[12]. The software provided regression coefficients and quadratic model for each parameter. The general formula was as follows (Equation 4):

$$Y = \beta_0 + \beta_1 X_1 + \beta_2 X_2 + \beta_3 X_3 + \beta_{11} X_1^2 + \beta_{12} X_1 X_2 + \beta_{13} X_1 X_3 + \beta_{22} X_2^2 + \beta_{23} X_2 X_3 + \beta_{33} X_3^2 \quad (4)$$

Where Y was the predicted parameter (e.g., antioxidative capacity), $\beta_0$ was the regression coefficient constant for intercept, $\beta_{ii}$ were the regression coefficients for linear, quadratic and interaction terms, and $X_i$ the independent variables (ESM1 Table S1). After optimization, the AM and UD extracts were obtained according to the optimized parameters (45 °C, 70 % EtOH, 1 h for AM and 49 °C, 70 % EtOH, 1 h for UD) and used for the bioactivity assays with a comparison of aqueous extract versions. The TCa, TCh, TPC and antioxidant capacity results of the optimal extracts were calculated according to the specific formula and coefficients of the parameters (ESM2 Appendix Table 1 and 2).

**Effects on bacterial growth**

The optimized [70 % EtOH (AM70, UD70)] and aqueous extracts (AM0, UD0) as controls were tested (extract concentration 0.05 % in the well) on four potential pathogenic bacterial strains [*Listeria innocua* Serotype 6a (Spanish National Culture Collection 910, CECT910), *Escherichia coli* O44:H18 (strain 042/EAEC), *Staphylococcus aureus* (CECT 86) and *Salmonella enterica enterica* Serovar *Thyphi* (CECT 4138)] and two potentially beneficial strains [*Lactocaseibacillus casei* (strain BL23, CECT 5275) and *Bifidobacterium animalis lactis* (strain Bb12)] that were cultivated in Brain Heart Infusion (BHI, Condalab, Spain) or De Man, Rogosa and Sharpe (MRS, Difco™ Lactobacilli MRS Broth, Ref. 288130) broths according to the ESM1 Table S2 at 37 °C for 1–3 days until a final optical density (OD) of 1.0 at 595 nm (Milton Roy Company Spectronic 20D) was obtained. The bacteria were inoculated at $OD_{595}$ = 1.0 in two-fold medium according to the need of each strain (ESM1 Table S2). The bacteria were centrifuged 5 min with 11 000 rpm with Eppendorf centrifuge 5424 (Eppendorf AG, Germany) to remove the medium and dilute the bacteria to the mentioned concentration ($OD_{595}$ = 1). To test the effects of ethanolic and aqueous AM and UD extracts on bacterial growth, a screening test using *E. coli* was performed. The extracts and controls were diluted in a range of 1/10 – 1/40. With the 1/20 dilution, the EtOH control did not have a significant effect on bacterial growth kinetics but the samples did, and therefore the 1/20 dilution was selected for further tests. The bacterial growth was monitored in presence or absence of 100 µl of the diluted different extracts or controls. Changes in OD at 595 nm of 1.0 were registered and strain's growth curves for the bacteria were attained in a 96-well microtiter plate reader SPECTROstar$^{Nano}$ (BMG LABTECH) measuring the absorbance at 37 ºC at 595 nm for 50 cycles, 1800 s per cycle. For *L. casei* and *B. lactis* the wells were sealed with 100 µl of vaseline oil (pure, pharma grade, PanReac AppliChem ITW Reagents) that was sterilized at 100 °C for 15 min (AccuBlock™ Digital Dry Bath, Labnet International, Inc.) and let to cool before addition, and the plates were sealed with Parafilm© M (Sigma, USA). The diluted samples and controls were stored in aliquots at –20 °C and thawed at RT before adding to the plate. Antimicrobial blend (AB, type 238.001, batch 281001, FLAVEX Naturextrakte, Germany) was used as a positive control as 1 % concentration that was diluted the same way as the samples with 70 % EtOH (AB70) or sterile water (AB0). Each extract sample was measured with five and each control with four replicates on the plate.

***ESM1 Table S2*** *The used bacterial strains, the culture medium and conditions used for each measurement.*

| Bacterial strain | Collection number | Culture medium | Condition | Gram (+/–) |
|---|---|---|---|---|
| *Listeria innocua* Serotype 6a | **CECT 910** | **BHI** | **Aerobic** | + |
| *Escherichia coli* O44:H18 | **Strain 042/EAEC** | **BHI** | **Aerobic** | – |
| *Staphylococcus aureus* | **CECT 86** | **BHI** | **Aerobic** | + |
| *Salmonella enterica* subsp. *enterica* Serovar *Thyphi* | **CECT 4138** | **BHI** | **Aerobic** | – |
| *Lacticaseibacillus casei* | **Strain BL23 CECT 5275** | **MRS** | **Anaerobic** | + |
| *Bifidobacterium animalis* subsp. *lactis* | **Strain Bb12** | **MRS** | **Anaerobic** | + |

*CECT: Spanish National Culture Collection (www.cect.org).*

*BHI: Condalab Brain Heart Infusion broth, MRS: Difco™ Lactobacilli De Man, Rogosa and Sharpe broth.*

*Temperature and time of growth for each bacterium were 37 °C for 24.5 hours.*

*The anaerobic conditions were made with sealing the wells with vaseline oil and parafilm.*

*Gram positive (+) and negative (–) indicate the gram staining characteristic of each bacterium.*

*Culture strains were stored at –80 °C with 50 % of glycerol in cryovials.*

The final values were corrected by omitting the baseline values of medium without bacteria. Then, the bacterial growth kinetics in presence and absence of the extracts were calculated in GraphPad Prism 5 software with modified Gompertz equation [13] as previously in Abdelkebir *et al.* [14] (Equation 5):

$$y = NO + C \times exp\{-exp[(2.718 \times mue/C) \times (Lag - x) + 1]\} \quad (5)$$

Where $y$ is the extent of growth as log colony-forming unit (CFU)/ml at time (h), *NO* the initial number of the cells as log CFU/ml, *C* the difference between initial and final cell numbers as log CFU/ml, the *exp* is exponent, *mue* the maximun specific growth rate as log CFU/ml/h and *Lag* the lenght of the lag phase as $h$. If the curve was unfit for Gompertz (the R below 0.85 or error in software), the values were calculated in Microsoft Excel (Microsoft Corporation, WA, USA) by hand with following way: *C = the final value of the sample – an average of all initial values on that plate*; *mue = the slope of the state of the exponential growth*, *lag = the time before the exponential growth starts*.

**Antiviral activity**

Antiviral effects of the AM and UD hydroethanolic and aqueous extracts (10 % v/v) were tested on murine macrophage cell line RAW 264.7 with murine noro virus (MNV) [15]. Because of the possible cytotoxic effects of EtOH, the extracts were diluted into 1/10 dilutions and mixed equally with MNV to reach titers of 5 log $TCID_{50}$ followed by an overnight incubation at 37 °C in shaking water bath (180 rpm). MNV with phosphate-buffered saline (PBS) and 7 % EtOH was also treated the same way and were used as untreated controls. The extracts were made in triplicates. After the incubation, 10-fold dilutions of each extract and controls were made (from $10^{-1}$ to $10^{-6}$) by serial dilutions. Subsequently, 20 µl of these diluted extracts or controls was added to the wells of RAW 264.7 monolayer in 96-well plates following an addition of 120 µl of Dulbecco's Modified Eagle's Medium High Glucose cell medium with 2% serum. The plates were incubated for 2 days at 37 °C, at 5 % of $CO_2$. The cells that were infected by the MNV were decayed and the number of wells with decayed cells was counted by checking with an optical microscope (Olympus CKX41). The virus concentration was obtained by Spearman-Karber method [16] by counting as $\log_{10}(N_x/N_0)$, where $N_x$ is the wells infected with virus+extract or EtOH control and $N_0$ is the cell wells of positive control of PBS.

**Anti-inflammatory activity**

Toll-like receptor 4 (TLR4) activation was performed in reporter cells of commercial Human embryonic kidney (HEK) cells (HEK-Blue™ hTLR4, InvivoGen) that were co-transfected to express the human TLR4, MD-2 and CD14 co-receptor genes, and an inducible SEAP (secreted embryonic alkaline phosphatase) reporter gene into HEK293 cells. NF-κB activation was assessed in human colon tumorigenic cell line HT-29 cells established in the Laboratory of Lactic Acid Bacteria and Probiotics (IATA-CSIC) (NFKβ-HT-29 reporter cells). The both cell lines were altered to contain a pNiFty2-secreted alkaline phosphatase (SEAP) plasmid (Invitrogen, Carlsbad, CA, USA). The cell culture and SEAP assay was conducted as described previously [17] with the exception that LPS was used as an inflammatory stimulant for both cell lines. The HEK-Blue™ hTLR4 cells were maintained in DMEM High Glucose and seeded in 96-well plates as 25.000 per well, grown for 24 h and washed with PBS prior to the experiment. AM and UD extraction samples and 70 % EtOH control were diluted to 1/10, 1/20 and 1/40. The sample and EtOH control dilutions (100 µl) were added to the plate in triplicate. Lipopolysaccharide from *Escherichia coli* 055:B5 (Sigma) at 10 ng/ml (LPS) was used as a positive inducer added simultaneously to AM and UD extracts (n=3). The plates were incubated for 16–18 h and SEAP activity was measured in cell culture media from each well using *p*-nitrophenyl phosphate according to manufacturer's instructions (Thermo Fisher Scientist, Waltham,

USA). The signal was quantified using a Spectrostar Nano microplate reader (CLARIOstar Plus BMG LABTECH) at 405 nm.

Then, cell viability was measured with a resazurin assay [18]. The supernatants were removed and replaced with 100 µl of resazurin (50 µg/ml) per well, then the plates were incubated at 37 °C for three hours and fluorescein was measured with CLARIOstar Plus (BMG Labtech, Ortenberg, Germany) at excitation/emission wavelengths of 540/590 nm. The results from SEAP were divided by resazurin to obtain arbitrary units (AU).

**Data analysis**

The statistical differences at level of 0.05 between extracts were analysed with SPSS statistics software (version 26, IBM) by using one-way analysis of variance (ANOVA). The Levene's test was used to evaluate the homogeneity of variance and Tukey or Tamhane's T2 post-hoc test was used according to the result from Levene to identify the differences and homogenous subsets. With non-normal data the Kruskal-Wallis was used with Mann-Whitney U-test corrected with Bonferroni to find statistical significances. Tamhane was used as a post hoc for the antibacterial effects. For the cell viability and TLR4 activation, the T-Test was used to find differences of the extracts on controls. The antiviral data was analysed with STATISTICA software version 7 (StatSoft Inc., Tulsa, OK, USA) and by using ANOVA followed by a pairwise comparison with the post-hoc Tukey's test. All the data were made as triplicates and presented as mean values with standard deviation.

## References


[1] Barba FJ, Grimi N, Vorobiev, E (2015) Evaluating the potential of cell disruption technologies for green selective extraction of antioxidant compounds from *Stevia rebaudiana* Bertoni leaves. J Food Engineering, 149:222–228. https://doi.org/10.1016/j.jfoodeng.2014.10.028

[2] von Wettstein D (1957) Chlorophyll-letale und der submikroskopische Formwechsel der Plastiden. Exp Cell Res 12(3):427–506. https://doi.org/10.1016/0014-4827(57)90165-9

[3] Singleton VL, Rossi JA (1965) Colorimetry of Total Phenolics with Phosphomolybdic-Phosphotungstic Acid Reagents. Am J Enol Vitic 16(3):144 LP – 158

[4] Re R, Pellegrini N, Proteggente A, Pannala A, Yang M, Rice-Evans C (1999) Antioxidant activity applying an improved ABTS radical cation decolorization assay. Free Radic Biol Med 26(9–10): 1231–1237. https://doi.org/10.1016/S0891-5849(98)00315-3

[5] Barba FJ, Criado MN, Belda-Galbis CM, Esteve MJ, Rodrigo D (2014) *Stevia rebaudiana* Bertoni as a natural antioxidant/antimicrobial for high pressure processed fruit extract:

Processing parameter optimization. *Food Chem* 148:261–267. https://doi.org/10.1016/j.foodchem.2013.10.048

[6] Ou B, Hampsch-Woodill M, Prior RL (2001) Development and validation of an improved oxygen radical absorbance capacity assay using fluorescein as the fluorescent probe. J Agric Food Chem 49(10): 4619–4626. https://doi.org/10.1021/jf010586o

[7] Daglia M (2012) Polyphenols as antimicrobial agents. Curr Opin Biotechnol 23(2): 174–181. https://doi.org/10.1016/j.copbio.2011.08.007

[8] Ali SI, Gopalakrishnan B, Venkatesalu V (2017) Pharmacognosy, Phytochemistry and Pharmacological Properties of *Achillea millefolium* L .: A Review. Phytother Res 31: 1140–1161. https://doi.org/10.1002/ptr.5840

[9] Zenão S, Aires A, Dias C, Saavedra MJ, Fernandes C (2017) Antibacterial potential of *Urtica dioica* and *Lavandula angustifolia* extracts against methicillin resistant *Staphylococcus aureus* isolated from diabetic foot ulcers. J Herb Med 10: 1–7. https://doi.org/10.1016/j.hermed.2017.05.003

[10] Altemimi A, Lakhssassi N, Baharlouei A, Watson DG, Lightfoot DA (2017) Phytochemicals: Extraction, isolation, and identification of bioactive compounds from plant extracts. Plants 6. https://doi.org/10.3390/plants6040042

[11] Das Q, Islam MR, Marcone MF, Warriner K, Diarra MS (2017) Potential of berry extracts to control foodborne pathogens. Food Control 73: 650–662. https://doi.org/10.1016/j.foodcont.2016.09.019

[12] Veiga M, Costa EM, Silva S, Pintado M (2020) Impact of plant extracts upon human health: A review. Crit Rev Food Sci Nutr 60(5): 873–886. https://doi.org/10.1080/10408398.2018.1540969

[13] Zwietering MH, Jongenburger I, Rombouts FM, Van't Riet K (1990) Modeling of the bacterial growth curve. Appl Environ Microbiol 56(6): 1875–1881. https://doi.org/10.1128/aem.56.6.1875-1881.1990

[14] Abdelkebir R et al. (2018) Effect of ultrasound technology combined with binary mixtures of ethanol and water on antibacterial and antiviral activities of *Erodium glaucophyllum* extracts. Innov Food Sci Emerg Technol 52: 189–196. https://doi.org/10.1016/j.ifset.2018.12.009

[15] Falcó I, Randazzo W, Gómez-Mascaraque L, Aznar R, López-Rubio A, Sánchez G (2017) Effect of (−)-epigallocatechin gallate at different pH conditions on enteric viruses. LWT 81: 250–257. https://doi.org/10.1016/j.lwt.2017.03.050

[16] Pintó RM, Diez JM, Bosch A (1994) Use of the colonic carcinoma cell line CaCo-2 for in vivo amplification and detection of enteric viruses. J Med Virol 44(3): 310–315. https://doi.org/10.1002/jmv.1890440317

[17] Munekata PES et al. (2020) Impact of ultrasound-assisted extraction and solvent composition on bioactive compounds and in vitro biological activities of thyme and rosemary. Food Res Int 134: 109242. https://doi.org/10.1016/J.FOODRES.2020.109242

[18] Calatayud M, Devesa V, Vélez D (2013) Differential toxicity and gene expression in Caco-2 cells exposed to arsenic species. Toxicol Lett 218(1): 70–80. https://doi.org/10.1016/j.toxlet.2013.01.013
