# Supplementary material for: In Vitro Bioactivities of Food Grade Extracts from Yarrow (Achillea millefolium L.) and Stinging Nettle (Urtica dioica L.) Leaves
Source: Plant Foods Hum Nutr. 2022 Nov 12;78(1):132–8. doi: 10.1007/s11130-022-01020-y (PMC9947014; doi:10.1007/s11130-022-01020-y)
Supplement: Supplementary file 2 — Supplementary file2 (PDF 833 KB) [file 11130_2022_1020_MOESM2_ESM.pdf]

ESM 2

In vitro bioactivities of food grade extracts from yarrow (*Achillea millefolium* L.) and stinging nettle (*Urtica dioica* L.) leaves

Plant Foods for Human Nutrition

Enni Mannila[a] (ORCID 0000-0002-8199-8137), Francisco J. Marti-Quijal[b] (ORCID 0000-0001-9034-5325), Marta Selma Royo[c] (ORCID 0000-0002-4258-947X), Marta Calatayud[c] (ORCID 0000-0003-3592-3377), Irene Falcó[c] (ORCID 0000-0002-4036-3274), Beatriz de la Fuente[b] (ORCID 0000-0002-4157-6159), Francisco J. Barba[b] (ORCID 0000-0002-5630-3989), Maria Carmen Collado[c,*] (ORCID 0000-0002-6204-4864) & Kaisa M. Linderborg[a,*] (ORCID 0000-0003-1977-7322)

[a]Food Sciences, Department of Life Technologies, University of Turku, Turku, Finland
[b]Nutrition and Food Science Area, Preventive Medicine and Public Health, Food Science, Toxicology and Forensic Medicine Department, Faculty of Pharmacy, Universitat de València, Avda. Vicent Andrés Estellés, s/n, 46100 Burjassot, València, Spain
[c]Institute of Agrochemistry and Food Technology-National Research Council (IATA-CSIC), Agustin Escardino 7, 46980 Paterna, Valencia, Spain
[*]Corresponding authors Maria Carmen Collado (mcolam@iata.csic.es) and Kaisa M. Linderborg (kaisa.linderborg@utu.fi)

Electronic Supplementary Material 2: Extraction optimization with Response Surface Methodology of *A. millefolium* L. and *U. dioica* L. with the quadratic functions

Results and Discussion for the Optimization of the Extraction

Relationships between the response and the extraction parameters are visualized in three-dimensional surface plots obtained from RSM in ESM2 Fig. S1. The extraction parameters significantly influenced the compositions of the extracts, and the optimal conditions for extraction were found when 45 °C, 70 % ethanol (EtOH) and 1 hour were used for *Achillea millefolium* L. (AM) and 49 °C, 70 % EtOH and 1 hour for *Urtica dioica* L. (UD). The measured total carotenoids (TCa), total chlorophylls (TCh), total phenolic compounds (TPC) and antioxidant capacities are represented for each parameter setting of the Box-Behnken design in ESM2 Tables S1–S2.

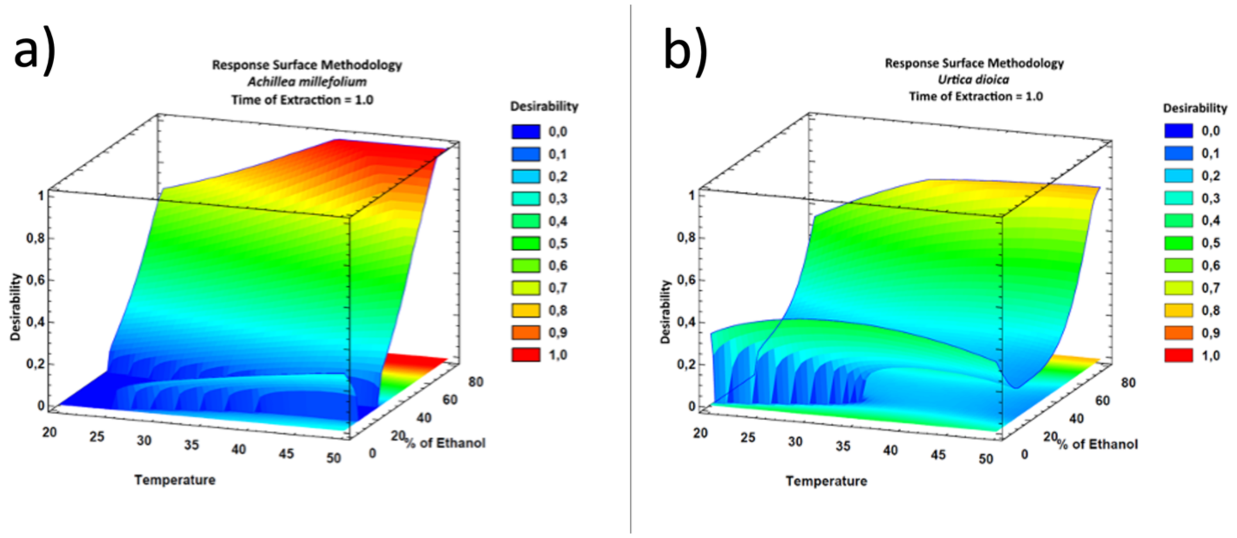

ESM2 Fig. S1 *The Response Surface Methodology graph of a) A. millefolium and b) U. dioica extraction optimization. The best extraction conditions were 1 hour with 70 % ethanol at 45 °C for AM and at 49 °C for UD. The red colour (Desirability 1,0) refers to high and blue (Desirability 0,0) to low desirability of the extract.*

ESM2 Table S1 *Total chlorophyll a and b (TChA, TChB), carotenoids (TCaC), phenolic content (TPC) and antioxidant capacity (TEAC, ORAC) of A. millefolium. Data represent means with standard deviation (n = 3).*

			A. millefolium					
Temperature (°C)	Ethanol (%)	Time (h)	TCaC (mg/L)	TChA (mg/L)	TChB (mg/L)	TPC (mg GAE/L)	TEAC (mM TE)	ORAC (mM TE)
20	0	1	15.79 ± 0.25	10.25 ± 0.18	21.11 ± 0.47	1542.14 ± 11.11	1.85 ± 0.19	10.85 ± 0.25
		24	18.48 ± 0.08	12.5 ± 0.45	23.54 ± 0.88	1431.79 ± 35.83	1.71 ± 0.19	9.10 ± 0.40
	35	12.5	10.17 ± 0.07	7.29 ± 0.14	8.58 ± 0.30	843.70 ± 2.10	1.54 ± 0.01	13.80 ± 1.00
	70	1	33.22 ± 0.48	90.38 ± 0.37	29.41 ± 0.74	1659.76 ± 8.40	6.17 ± 0.29	33.35 ± 0.85
		24	30.89 ± 0.43	110.64 ± 1.22	36.90 ± 1.46	1651.27 ± 24.76	7.90 ± 0.68	32.10 ± 0.75
35	0	12.5	18.27 ± 0.07	8.91 ± 0.21	17.63 ± 0.34	1402.69 ± 15.86	2.48 ± 0.02	8.55 ± 0.35
	35	1	8.10 ± 0.06	12.85 ± 0.21	11.53 ± 0.33	2202.98 ± 19.25	7.20 ± 0.68	32.90 ± 2.05
35		12.5	12.44 ± 0.62[a]	7.80 ± 0.80[a]	9.03 ± 0.68[a]	1005.58 ± 50.59[a]	2.42 ± 0.55[a]	13.60 ± 2.45[a]
		24	12.17 ± 0.08	7.08 ± 0.25	8.76 ± 0.40	957.68 ± 20.03	2.04 ± 0.20	14.80 ± 0.70
	70	12.5	30.77 ± 0.02	109.22 ± 0.20	37.22 ± 0.39	1733.72 ± 16.67	8.82 ± 0.80	32.60 ± 2.90
50	0	1	13.04 ± 0.19	7.87 ± 0.18	15.78 ± 0.22	1724.02 ± 39.24	3.12 ± 0.36	13.50 ± 0.50
		24	11.19 ± 0.06	7.87 ± 0.18	10.54 ± 0.79	1116.53 ± 9.15	1.86 ± 0.21	7.20 ± 0.65
	35	12.5	10.66 ± 0.03	5.38 ± 0.09	5.16 ± 0.30	2131.44 ± 14.70	8.94 ± 0.19	35.35 ± 1.15
	70	1	40.33 ± 0.12	110.13 ± 0.70	39.66 ± 1.26	2247.85 ± 8.40	9.16 ± 0.47	35.80 ± 2.10
		24	28.35 ± 0.74	55.24 ± 0.78	19.43 ± 1.72	2018.67 ± 16.40	7.34 ± 0.35	35.75 ± 1.45

[a] Average of the duplicate samples. TCaC: Total carotenoid content, TChA: Total chlorophyll a content, TChB: Total chlorophyll b content, TPC: Total phenolic content, TEAC: Trolox equivalent antioxidant capacity, ORAC: Oxygen radical antioxidant capacity. mg GAE/L: milligrams of gallic acid equivalents. mM TE: millimolar Trolox equivalents.

ESM2 Table S2 *Total chlorophyll a and b (TChA, TChB), carotenoids (TCaC), phenolic content (TPC) and antioxidant capacity (TEAC, ORAC) of U. dioica. Data represent means with standard deviation (n = 3).*

			U. dioica					
Temperature (°C)	Ethanol (%)	Time (h)	TCaC (mg/L)	TChA (mg/L)	TChB (mg/L)	TPC (mg GAE/L)	TEAC (mM TE)	ORAC (mM TE)
20	0	1	ND	93.85 ± 0.19	154.47 ± 0.77	1656.38 ± 8.25	2.93 ± 0.20	18.90 ± 0.45
		24	1.51 ± 0.24	57.39 ± 1.06	91.61 ± 2.50	1258.53 ± 9.52	1.86 ± 0.28	9.65 ± 0.55
	35	12.5	10.39 ± 0.04	7.49 ± 0.08	10.87 ± 0.20	824.30 ± 23.53	3.07 ± 0.16	12.00 ± 0.55
	70	1	45.11 ± 0.39	47.84 ± 1.29	26.30 ± 2.02	1321.89 ± 7.84	7.19 ± 0.26	26.80 ± 1.35
		24	49.34 ± 0.43	91.86 ± 0.33	39.54 ± 0.96	988.43 ± 12.59	3.29 ± 0.42	15.25 ± 1.05
35	0	12.5	10.17 ± 0.40	53.00 ± 0.82	91.46 ± 1.31	1365.52 ± 9.52	2.59 ± 0.04	11.20 ± 0.40
	35	1	9.76 ± 0.42	22.99 ± 0.43	38.88 ± 1.06	1290.72 ± 7.84	2.83 ± 0.05	21.35 ± 1.20
35		12.5	6.50 ± 0.16[a]	17.05 ± 0.54[a]	25.08 ± 0.78[a]	1250.21 ± 13.22[a]	5.05 ± 0.02[b]	22.45 ± 1.05[b]
		24	9.11 ± 0.07	8.40 ± 0.17	11.06 ± 0.26	851.30 ± 12.97	1.85 ± 0.12	15.60 ± 1.00
	70	12.5	62.36 ± 1.11	83.78 ± 1.43	42.33 ± 2.37	1335.39 ± 3.12	7.89 ± 0.00	21.05 ± 1.65
50	0	1	5.55 ± 0.06	54.76 ± 0.42	91.88 ± 0.44	1511.99 ± 6.49	2.65 ± 0.04	15.65 ± 0.65
		24	8.37 ± 0.11	10.19 ± 0.21	18.98 ± 0.23	1343.70 ± 9.52	3.69 ± 0.48	13.10 ± 0.90
	35	12.5	7.39 ± 0.09	15.62 ± 0.12	24.32 ± 0.13	1197.23 ± 25.19	2.73 ± 0.25	19.05 ± 1.15
	70	1	61.86 ± 0.79	72.09 ± 1.61	30.28 ± 2.49	1570.16 ± 3.60	10.02 ± 0.35	27.20 ± 0.60
		24	62.69 ± 0.18	84.46 ± 0.34	50.38 ± 0.66	1184.76 ± 6.49	7.01 ± 0.35	17.85 ± 1.00

[a] Average of the sample 10 made as duplicate. [b] Average of the sample 10. TCaC: Total carotenoid content, TChA: Total chlorophyll a content, TChB: Total chlorophyll b content, TPC: Total phenolic content, TEAC: Trolox equivalent antioxidant capacity, ORAC: Oxygen radical antioxidant capacity. mg GAE/L: milligrams of gallic acid equivalents. mM TE: millimolar Trolox equivalents. ND: Not detected.

Calculated according to the quadratic functions (ESM2 Appendix Tables 1 and 2), TCa, TCh, TPC and antioxidant capacities of extracts obtained under optimal conditions as well as those of aqueous extracts are shown in ESM2 Table S3.

ESM2 Table S3 *Total carotenoids, chlorophylls, phenolic compounds and antioxidant capacities calculated according to the regression coefficients and quadratic models for the optimized extracts in 70 % ethanol and their aqueous versions.*

		TCa (mg/L)	TChA (mg/L)	TChB (mg/L)	TPC (mg GAE/L)	TEAC (mM TE)	ORAC (mM TE)
AM	70 % ethanol	37.6	106.4	38.6	2318.3	9.4	38.0
	aqueous	14.0	16.0	19.5	1838.0	3.9	14.5
UD	70 % ethanol	60.7	73.3	37.4	1575.1	9.5	27.0
	aqueous	5.6	61.4	96.6	1566.4	2.3	16.5

AM: A. millefolium, UD: U. dioica, TCa: Total carotenoid content, TChA: Total chlorophyll a content, TChB: Total chlorophyll b content, TPC: Total phenolic content, TEAC: Trolox equivalent antioxidant capacity, ORAC: Oxygen radical antioxidant capacity. mg GAE/L: milligrams of gallic acid equivalents. mM TE: millimolar Trolox equivalents. See ESM2 Appendix tables for the used quadratic functions.

When the experimental values of the extracts that were extracted with close to optimal parameters (+50°C, 1 hour and either in 70 or 0 % EtOH, ESM2 Table S1–S2) were compared to the calculated optimal extracts (ESM2 Table S3), the results were very similar (e.g. TPC of UD measured value in 70 % EtOH: 1570.16 ± 3.60, calculated value: 1575.10 mg GAE/L). This indicates that the equations from optimization give reliable values for the TCa, TCh and antioxidant capacities and these can be calculated consistently also with differing parameters. The predicted values from RSM have been found to be very similar to experimental values reported previously [1, 2]. Extraction of AM and UD have been optimized with different methods, and here, the optimal time (1 h) is less for AM and longer for UD than in previous literature [1, 3, 4]. The obtained temperatures of 45 and 49 °C here are reasonable, since temperature of 40 °C has previously noted to be good for obtaining spinach extracts high in carotenoids [2]. EtOH concentration of 70 % is rather high, but in accordance with a previous study [3]. Methanol has found to extract more phenolic compounds than EtOH [1, 4], but since it is not a food-grade solvent it was not chosen for the present study.

The obtained TPC is quite small compared to the previous studies where the TPC of AM has been 182.92±1.72 mg GAE/g [3] and TPC of UD 460.13±8.25 mg GAE/g [4]. However, Dokhani *et al.*

[5] found similar TPC of AM (32.7±3.6 mg/g dw) and Vajić *et al.* [1] optimized maximal phenolic yield of UD leaves for 9.9±0.3 and 12.6±0.5 mg GAE/g dry weight for maceration and ultrasound assisted extraction, respectively. The present results are in accordance with previous optimization study from our research group with *S. rebaudiana* Bertoni where extract of 1.25 % *Stevia* concentration gave TPC of 2261.13±41.74 mg GAE/L, TEAC of 16.62±0.21 mM TE and ORAC of 16.91±0.83 mM TE [6]. TEAC values were relatively low compared to those of *S. rebaudiana* study, but TEAC of AM has also previously measured to be in the range of 4.18–12.3 mM [7]. The plants of the present study are from early season and possibly the TPC and corresponding antioxidant activity has not risen to the same levels as in more mature plant materials in previous studies.

The plants of the present study are from early season since young leaves are preferred for food use and early season UD contains higher amounts of bioactive compounds, such as carotenoids and chlorophylls, than older leaves [8–10]. Repajić *et al.* [10] found that UD leaves collected before flowering stage contained the highest amounts of bioactive compounds (TCa 61.46±0.08, TCh 1126.94±0.66 mg/100 g dm) and antioxidant capacity (ORAC 11.96±0.02 mmol TE/100g dm). In our study, the used young leaves of UD contain higher amounts of TPC and ORAC values than UD harvested in July and in October in a previous study [11]. In addition to the extraction techniques, the harvesting time affects the composition of the extracts.

References

[1] Vajić UJ, Grujić-Milanović J, Živković J, Šavikin K, Godevac D, Miloradović Z, Bugarski B, Mihailović-Stanojević N (2015) Optimization of extraction of stinging nettle leaf phenolic compounds using response surface methodology. Ind Crops Prod 74: 912–917. https://doi.org/10.1016/j.indcrop.2015.06.032

[2] Altemimi A, Lightfoot DA, Kinsel M, Watson DG (2015) Employing response surface methodology for the optimization of ultrasound assisted extraction of lutein and β-carotene from spinach. Molecules 20: 6611–6625. https://doi.org/10.3390/molecules20046611

[3] Milutinović M, Radovanović N, Ćorović M, Šiler-Marinković S, Rajilić-Stojanović M, Dimitrijević-Branković S (2015) Optimisation of microwave-assisted extraction parameters for antioxidants from waste *Achillea millefolium* dust. Ind Crops Prod 77: 333–341. https://doi.org/10.1016/j.indcrop.2015.09.007

[4] Stanojević LP, Stanković MZ, Cvetković DJ, Cakić MD, Ilić DP, Nikolić VD, Stanojević JS (2016) The effect of extraction techniques on yield, extraction kinetics, and antioxidant activity of aqueous-methanolic extracts from nettle (*Urtica dioica* L.) leaves. Sep Sci Technol 51: 1817–1829. https://doi.org/10.1080/01496395.2016.1178774

[5] Dokhani S, Cottrell T, Khajeddin J, Mazza G (2005) Analysis of aroma and phenolic components of selected achillea species. *Plant Foods Hum Nutr* 60: 55–62. https://doi.org/10.1007/s11130-005-5100-9

[6] Barba FJ, Criado MN, Belda-Galbis CM, Esteve MJ, Rodrigo D (2014) *Stevia rebaudiana* Bertoni as a natural antioxidant/antimicrobial for high pressure processed fruit extract: Processing parameter optimization. *Food Chem* 148:261–267. https://doi.org/10.1016/j.foodchem.2013.10.048

[7] Saeidnia S, Gohari AR, Mokhber-Dezfuli N, Kiuchi F (2011) A review on phytochemistry and medicinal properties of the genus *Achillea*. DARU J Pharm Sci 19: 173–186.

[8] Dhouibi R et al. (2020) Screening of pharmacological uses of *Urtica dioica* and others benefits. Prog Biophys Mol Biol 150: 67–77. https://doi.org/10.1016/j.pbiomolbio.2019.05.008

[9] Kukrić ZZ, Topalić-Trivunović LN, Kukavica BM, Matoš SB, Pavičić SS, Boroja MM, Savić AV (2012) Characterization of antioxidant and antimicrobial activities of nettle leaves (*Urtica dioica* L.). APTEFF 43: 257–272. https://doi.org/10.2298/APT1243257K

[10] Repajić M et al. (2021) Bioactive compounds in wild nettle (*Urtica dioica* l.) leaves and stalks: Polyphenols and pigments upon seasonal and habitat variations. *Foods* 10(1). https://doi.org/10.3390/foods10010190

[11] Tian Y, Puganen A, Alakomi HL, Uusitupa A, Saarela M, Yang B (2018) Antioxidative and antibacterial activities of aqueous ethanol extracts of berries, leaves, and branches of berry plants. Food Res Int 106: 291–303. https://doi.org/10.1016/j.foodres.2017.12.071

ESM2 Appendix: Quadratic functions

***Achillea millefolium* L.**

ESM2 Appendix Table 1 Coefficients of regression for *Achillea millefolium* L.

	TCa	TChA	TChB	TPC	TEAC	ORAC
constant	12,6904	-38,9023	3,73074	1,82572	1215,91	4341,91
A (1): Temperature	0,124947	2,30458	0,942647	-0,0160348	-24,6875	-143,21
B (2): % of Ethanol	-0,593236	-1,12569	-0,850365	-0,0146188	19,8102	67,9483
C (3): Time of extraction	0,587799	2,38248	0,778356	-0,04042	82,0811	-126,287
AA (11)	-0,00216452	-0,0239804	-0,0129694	0,000393257	1,90825	2,54846
AB (12)	0,00348095	-0,00615595	0,00264286	0,000259238	0,241486	0,249315
AC (13)	-0,0102841	-0,058092	-0,0256406	-0,000520145	-3,38541	-0,482231
BB (22)	0,0111175	0,0386412	0,0143979	0,000138068	0,680616	-0,187514
BC (23)	-0,00470807	-0,010591	-0,0030795	0,00014913	0,407518	0,418825
CC (33)	-0,00579219	-0,0133768	0,00269474	0,00137076	-1,42387	3,23268

Carotenoids (TCa) = 12,6904 + 0,124947*Temperature - 0,593236*% of Ethanol + 0,587799*Time of extraction - 0,00216452*Temperature^2 + 0,00348095*Temperature*% of Ethanol - 0,0102841*Temperature*Time of extraction + 0,0111175*% of Ethanol^2 - 0,00470807*% of Ethanol*Time of extraction - 0,00579219*Time of extraction^2

Chlorophyll a (TChA) = -38,9023 + 2,30458*Temperature - 1,12569*% of Ethanol + 2,38248*Time of extraction - 0,0239804*Temperature^2 - 0,00615595 *Temperature*% of Ethanol

- 0,058092*Temperature*Time of extraction + 0,0386412*% of Ethanol^2 - 0,010591*% of Ethanol*Time of extraction - 0,0133768*Time of extraction^2

Chlorophyll b (TChB) = 3,73074 + 0,942647*Temperature - 0,850365*% of Ethanol + 0,778356*Time of extraction - 0,0129694*Temperature^2 + 0,00264286*Temperature*% of Ethanol - 0,0256406*Temperature*Time of extraction + 0,0143979*% of Ethanol^2 - 0,0030795*% of Ethanol*Time of extraction + 0,00269474*Time of extraction^2

Total Phenolic Content (TPC) = 1,82572 - 0,0160348*Temperature - 0,0146188*% of Ethanol - 0,04042*Time of extraction + 0,000393257*Temperature^2 + 0,000259238 *Temperature*% of Ethanol - 0,000520145*Temperature*Time of extraction + 0,000138068*% of Ethanol^2 + 0,00014913*% of Ethanol*Time of extraction + 0,00137076*Time of extraction^2

Trolox Equivalent Antioxidative Capacity (TEAC) = 1215,91 - 24,6875*Temperature + 19,8102*% of Ethanol + 82,0811*Time of extraction + 1,90825*Temperature^2 + 0,241486 *Temperature*% of Ethanol - 3,38541*Temperature*Time of extraction + 0,680616*% of Ethanol^2 + 0,407518*% of Ethanol*Time of extraction - 1,42387*Time of extraction^2

Oxygen Radical Absorbance Capacity (ORAC) = 4341,91 - 143,21*Temperature + 67,9483*% of Ethanol - 126,287*Time of extraction + 2,54846*Temperature^2 + 0,249315 *Temperature*% of Ethanol - 0,482231*Temperature*Time of extraction - 0,187514*% of Ethanol^2 + 0,418825*% of Ethanol*Time of extraction + 3,23268*Time of extraction^2

***Urtica dioica* L.**

ESM2 Appendix Table 2 Coefficients of regression for *Urtica dioica* L.

	TCa	TChA	TChB	TPC	TEAC	ORAC
constant	-13,776	87,7501	167,273	1,31881	1540,64	2939,61
A (1): Temperature	0,972414	0,477568	-0,0733755	0,0193305	76,7675	46,2733
B (2): % of Ethanol	-0,829801	-4,06951	-5,12396	-0,0214302	-69,3772	28,2655
C (3): Time of extraction	0,527885	-0,76734	-3,15337	-0,0170292	92,9198	-170,873
AA (11)	-0,0119629	-0,0197945	-0,0265891	-0,000286904	-1,3431	-0,764456
AB (12)	0,00421214	0,0245579	0,0357276	0,000119976	1,19353	0,134491
AC (13)	-0,00152391	-0,028808	-0,0023029	0,000128768	2,17522	1,29268
BB (22)	0,0201464	0,0427606	0,035362	0,000224609	1,66023	-0,046264
BC (23)	0,000230124	0,0426748	0,0525199	-0,0000474224	-2,13359	-0,563597
CC (33)	-0,0162658	-0,0023649	0,0105289	-0,0000325403	-6,52717	3,14169

Carotenoids (TCa) = -13,776 + 0,972414*Temperature - 0,829801*% of Ethanol + 0,527885*Time of extraction - 0,0119629*Temperature^2 + 0,00421214*Temperature*% of Ethanol - 0,00152391*Temperature*Time of extraction + 0,0201464*% of Ethanol^2 + 0,000230124*% of Ethanol*Time of extraction - 0,0162658*Time of extraction^2

Chlorophyll a (TChA) = 87,7501 + 0,477568*Temperature - 4,06951*% of Ethanol - 0,76734*Time of extraction - 0,0197945*Temperature^2 + 0,0245579*Temperature*% of Ethanol - 0,028808*Temperature*Time of extraction + 0,0427606*% of Ethanol^2 + 0,0426748*% of Ethanol*Time of extraction - 0,0023649*Time of extraction^2

Chlorophyll b(TChB) = 167,273 - 0,0733755*Temperature - 5,12396*% of Ethanol - 3,15337*Time of extraction - 0,0265891*Temperature^2 + 0,0357276*Temperature*% of Ethanol - 0,0023029*Temperature*Time of extraction + 0,035362*% of Ethanol^2 + 0,0525199*% of Ethanol*Time of extraction + 0,0105289*Time of extraction^2

Total Phenolic Content (TPC) = 1,31881 + 0,0193305*Temperature - 0,0214302*% of Ethanol - 0,0170292*Time of extraction - 0,000286904*Temperature^2 + 0,000119976*Temperature*% of Ethanol + 0,000128768*Temperature*Time of extraction + 0,000224609*% of Ethanol^2 - 0,0000474224*% of Ethanol*Time of extraction - 0,0000325403*Time of extraction^2

Trolox Equivalent Antioxidative Capacity (TEAC) = 1540,64 + 76,7675*Temperature - 69,3772*% of Ethanol + 92,9198*Time of extraction - 1,3431*Temperature^2 + 1,19353*Temperature*% of Ethanol + 2,17522*Temperature*Time of extraction + 1,66023*% of Ethanol^2 - 2,13359*% of Ethanol*Time of extraction -6,52717*Time of extraction^2

Oxygen Radical Absorbance Capacity (ORAC) = 2939,61 + 46,2733*Temperature + 28,2655*% of Ethanol - 170,873*Time of extraction - 0,764456*Temperature^2 + 0,134491*Temperature*% of Ethanol + 1,29268*Temperature*Time of extraction - 0,046264*% of Ethanol^2 - 0,563597*% of Ethanol*Time of extraction + 3,14169*Time of extraction^2
