# Supplementary material for: In Vitro Bioactivities of Food Grade Extracts from Yarrow (Achillea millefolium L.) and Stinging Nettle (Urtica dioica L.) Leaves
Source: Plant Foods Hum Nutr. 2022 Nov 12;78(1):132–8. doi: 10.1007/s11130-022-01020-y (PMC9947014; doi:10.1007/s11130-022-01020-y)
Supplement: Supplementary file 3 — Supplementary file3 (PDF 232 KB) [file 11130_2022_1020_MOESM3_ESM.pdf]

ESM 3

In vitro bioactivities of food grade extracts from yarrow (*Achillea millefolium* L.) and stinging nettle (*Urtica dioica* L.) leaves

Plant Foods for Human Nutrition

Enni Mannila[a] (ORCID 0000-0002-8199-8137), Francisco J. Marti-Quijal[b] (ORCID 0000-0001-9034-5325), Marta Selma Royo[c] (ORCID 0000-0002-4258-947X), Marta Calatayud[c] (ORCID 0000-0003-3592-3377), Irene Falcó[c] (ORCID 0000-0002-4036-3274), Beatriz de la Fuente[b] (ORCID 0000-0002-4157-6159), Francisco J. Barba[b] (ORCID 0000-0002-5630-3989), Maria Carmen Collado[c,*] (ORCID 0000-0002-6204-4864) & Kaisa M. Linderborg[a,*] (ORCID 0000-0003-1977-7322)

[a]Food Sciences, Department of Life Technologies, University of Turku, Turku, Finland
[b]Nutrition and Food Science Area, Preventive Medicine and Public Health, Food Science, Toxicology and Forensic Medicine Department, Faculty of Pharmacy, Universitat de València, Avda. Vicent Andrés Estellés, s/n, 46100 Burjassot, València, Spain
[c]Institute of Agrochemistry and Food Technology-National Research Council (IATA-CSIC), Agustin Escardino 7, 46980 Paterna, Valencia, Spain
[*]Corresponding authors Maria Carmen Collado (mcolam@iata.csic.es) and Kaisa M. Linderborg (kaisa.linderborg@utu.fi)

Electronic Supplementary Material 3: Tables of the effects on pathogenic and beneficial bacteria

ESM3 Table S1 The effects of the extracts on the growth curve parameters on the pathogenic bacteria.

Condition	Composition	Growth rate [mue]	MOD [C]	Lag (h)
Listeria innocua				
Bacteria	Water	0.373±0.061	1.423±0.088	6.735±0.368
Bacteria + AB	Water	NG	NG	NG
Bacteria + AM	Water	0.389±0.066	1.169±0.045	6.524±0.657
Bacteria + UD	Water	0.327±0.026	1.394±0.084	5.142±0.345**
Bacteria	70% EtOH	0.268±0.037	1.310±0.187	12.315±0.444***
Bacteria + AB	70% EtOH	0.205±0.008	0.480±0.022	-0.097±0.078***
Bacteria + AM	70% EtOH	0.059±0.004*	1.043±0.142	12.562±0.465
Bacteria + UD	70% EtOH	0.067±0.006*	0.9438±0.120	9.608±0.300**
Escherichia coli				
Bacteria	Water	0.611±0.021	1.643±0.055	1.275±0.029
Bacteria + AB	Water	0.605±0.029	1.525±0.047	1.319±0.043
Bacteria + AM	Water	0.555±0.029	1.463±0.045	1.298±0.030
Bacteria + UD	Water	0.525±0.025	1.526±0.089	1.319±0.082

Bacteria	70% EtOH	0.298±0.010**	1.268±0.043*	1.352±0.044
Bacteria + AB	70% EtOH	0.233±0.004**	1.860±0.171	0.499±0.079***
Bacteria + AM	70% EtOH	0.317±0.042	1.394±0.083	1.212±0.129
Bacteria + UD	70% EtOH	0.456±0.033**	1.376±0.026	1.524±0.046
Staphylococcus aureus				
Bacteria	Water	0.356±0.004	2.410±0.101	1.522±0.201
Bacteria + AB	Water	NG	NG	NG
Bacteria + AM	Water	0.310±0.015*	2.154±0.126	1.853±0.227
Bacteria + UD	Water	0.642±0.029***	2.674±0.081	2.926±0.145**
Bacteria	70% EtOH	0.206±0.009***	1.680±0.181*	3.342±0.205***
Bacteria + AB	70% EtOH	0.034±0.002***	0.643±0.095**	0.000±0.000**
Bacteria + AM	70% EtOH	0.352±0.039*	3.024±0.710	4.778±0.821
Bacteria + UD	70% EtOH	0.346±0.044***	2.588±0.483	3.736±0.505
Salmonella enterica				
Bacteria	Water	0.970±0.067	2.450±0.023	1.890±0.109
Bacteria + AB	Water	1.00±0.091	2.140±0.022***	2.140±0.123
Bacteria + AM	Water	0.793±0.030	2.366±0.061	1.715±0.057
Bacteria + UD	Water	0.851±0.025	2.252±0.025***	1.843±0.045
Bacteria	70% EtOH	1.147±0.302	2.638±0.121	3.599±0.217**
Bacteria + AB	70% EtOH	0.082±0.108	1.720±0.880	5.296±0.408*
Bacteria + AM	70% EtOH	0.785±0.078	2.516±0.038	3.096±0.081
Bacteria + UD	70% EtOH	0.911±0.025	2.396±0.011	3.021±0.032

The values are presented as mean and standard deviation. The asterisks show significant differences between the control (bacteria in water or in EtOH) and each of the extract treatment inside the same composition: * $p < 0.05$, ** $p < 0.01$, *** $p < 0.001$. EtOH control is compared to the baseline (bacteria in water) to find effects originating from ethanol instead of the extracts and indicated with asterisks as mentioned above. AB: Antimicrobial blend as a positive control. AM: *A. millefolium*. UD: *U. dioica*. EtOH: Ethanol. mue: the maximun specific growth rate as log CFU/ml/h. MOD, C: Maximal optical density. Lag: the length of the lag phase as hours. NG: No growth.

ESM3 Table S2 The effects of the extracts on the growth curve parameters on the beneficial bacteria

Condition	Composition	Growth rate [mue]	MOD [C]	Lag (h)
Lacticaseibacillus casei				
Bacteria	Water	0.739±0.118	4.280±0.307	4.504±0.373
Bacteria + AB	Water	0.339±0.050	3.757±0.227	8.296±0.742*
Bacteria + AM	Water	0.644±0.010	4.628±0.026	3.775±0.054
Bacteria + UD	Water	0.767±0.036	4.628±0.178	4.422±0.139
Bacteria	70% EtOH	0.581±0.051	3.868±0.094	4.364±0.211
Bacteria + AB	70% EtOH	NG	NG	NG
Bacteria + AM	70% EtOH	0.475±0.054	4.826±0.572	3.587±0.476
Bacteria + UD	70% EtOH	0.511±0.086	4.390±0.622	3.999±0.650
Bifidobacterium lactis				
Bacteria	Water	0.346±0.090	3.353±0.455	10.645±0.277
Bacteria + AB	Water	0.050±0.003	1.128±0.141*	17.000±0.000**
Bacteria + AM	Water	0.276±0.064	3.228±0.675	10.363±0.448
Bacteria + UD	Water	0.303±0.062	3.054±0.381	10.878±0.236
Bacteria	70% EtOH	0.247±0.106	3.143±1.052	12.780±0.774
Bacteria + AB	70% EtOH	0.014±0.005	0.075±0.010	0.000±0.000**
Bacteria + AM	70% EtOH	0.260±0.082	2.848±0.055	12.882±0.392
Bacteria + UD	70% EtOH	0.237±0.069	3.274±0.462	11.970±1.105

The values are presented as mean and standard deviation. The asterisks show significant differences between the control (bacteria in water or in EtOH) and each of the extract treatment inside the same composition: * $p < 0.05$, ** $p < 0.01$, *** $p < 0.001$. AB: Antimicrobial blend as a positive control. AM: *A. millefolium*. UD: *U. dioica*. EtOH: Ethanol. mue: the maximun specific growth rate as log CFU/ml/h. MOD, C: Maximal optical density. Lag: the length of the lag phase as hours. NG: No growth.
