# Supplementary material for: In Vitro Bioactivities of Food Grade Extracts from Yarrow (Achillea millefolium L.) and Stinging Nettle (Urtica dioica L.) Leaves
Source: Plant Foods Hum Nutr. 2022 Nov 12;78(1):132–8. doi: 10.1007/s11130-022-01020-y (PMC9947014; doi:10.1007/s11130-022-01020-y)
Supplement: Supplementary file 4 — Supplementary file4 (PDF 1223 KB) [file 11130_2022_1020_MOESM4_ESM.pdf]

ESM 4

In vitro bioactivities of food grade extracts from yarrow (*Achillea millefolium* L.) and stinging nettle (*Urtica dioica* L.) leaves

Plant Foods for Human Nutrition

Enni Mannila[a] (ORCID 0000-0002-8199-8137), Francisco J. Marti-Quijal[b] (ORCID 0000-0001-9034-5325), Marta Selma Royo[c] (ORCID 0000-0002-4258-947X), Marta Calatayud[c] (ORCID 0000-0003-3592-3377), Irene Falcó[c] (ORCID 0000-0002-4036-3274), Beatriz de la Fuente[b] (ORCID 0000-0002-4157-6159), Francisco J. Barba[b] (ORCID 0000-0002-5630-3989), Maria Carmen Collado[c,*] (ORCID 0000-0002-6204-4864) & Kaisa M. Linderborg[a,*] (ORCID 0000-0003-1977-7322)

[a]Food Sciences, Department of Life Technologies, University of Turku, Turku, Finland
[b]Nutrition and Food Science Area, Preventive Medicine and Public Health, Food Science, Toxicology and Forensic Medicine Department, Faculty of Pharmacy, Universitat de València, Avda. Vicent Andrés Estellés, s/n, 46100 Burjassot, València, Spain
[c]Institute of Agrochemistry and Food Technology-National Research Council (IATA-CSIC), Agustin Escardino 7, 46980 Paterna, Valencia, Spain
[*]Corresponding authors Maria Carmen Collado (mcolam@iata.csic.es) and Kaisa M. Linderborg (kaisa.linderborg@utu.fi)

Electronic Supplementary Material 4: Cell viability of HEK-Blue™ hTLR4 cells and NFKβ-HT-29 reporter cell line

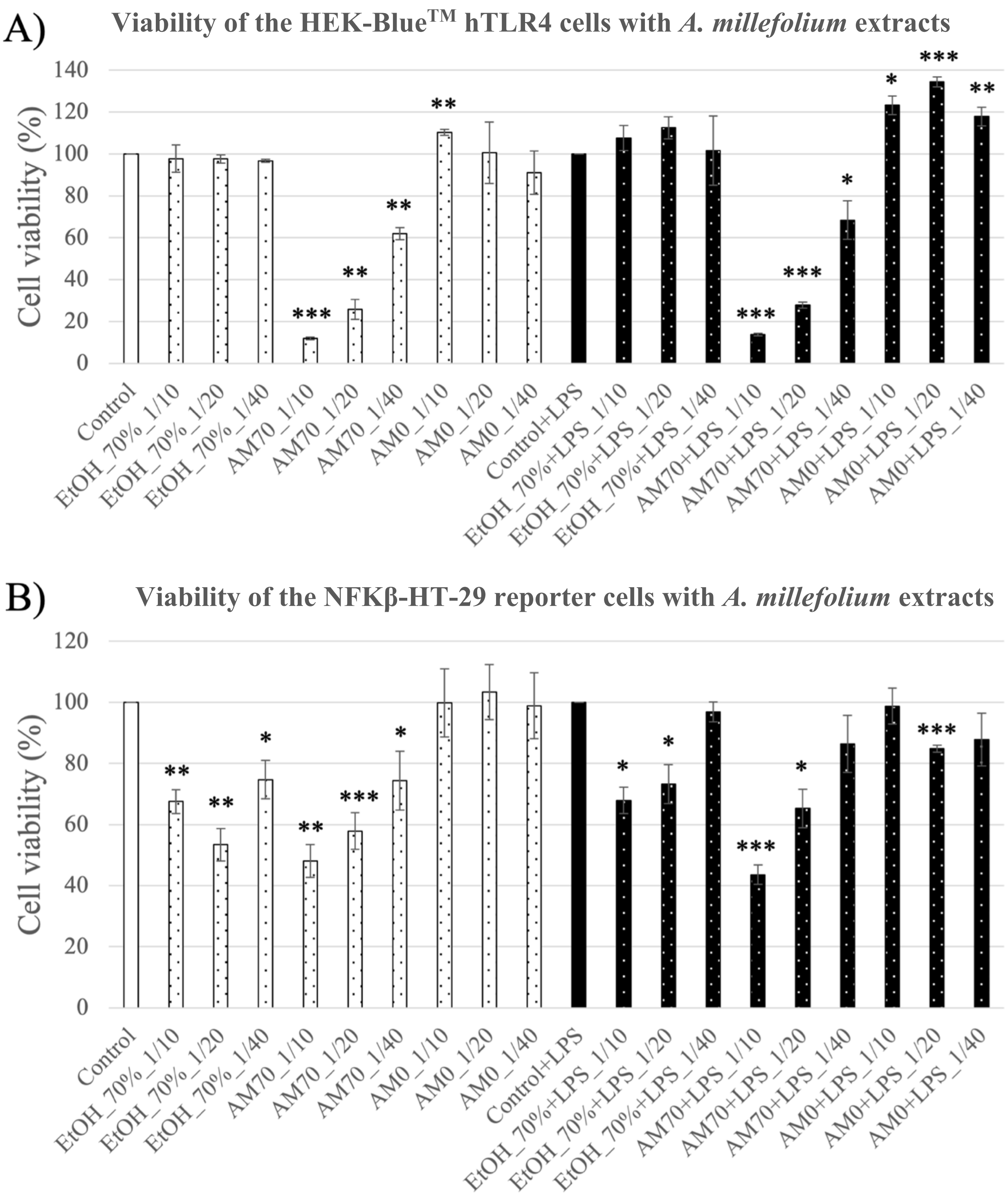

ESM4 Fig. S1 *Viability of HEK-Blue™ hTLR4 cells (A) and NFKβ-HT-29 reporter cell line (B) with different dilutions of A. millefolium extracts. Controls were set as 100% and the ethanol and extracts were compared to respective control (with or without LPS), the asterisks show significant differences: * p < 0.05, ** p < 0.01, *** p < 0.001. The control with LPS was 88 % of the Control in HEK-Blue™ hTLR4 and 109 % in NFKβ-HT-29 cells. Data represent means with standard deviation (n = 3). EtOH: Ethanol; AM: A. millefolium in 70 % Ethanol or 0 % as aqueous extract; LPS: Lipopolysaccharide.*

ESM4 Table S1 *The p values of cell controls compared with the ethanol controls and A. millefolium extracts for of HEK-Blue™ hTLR4 and NFKβ-HT-29 reporter cell viabilities (ESM4 Fig. S1). The bolded values show statistical significance at the level of 0.05 with a T-test. Values of lipopolysaccharide induction are inside brackets.*

	p values	
Extract	Control HEK-Blue™ hTLR4 (with LPS)	Control *NFKβ-HT-29* (with LPS)
EtOH 1/10 (+LPS)	0.579 (0.161)	**0.005 (0.037†)**
EtOH 1/20 (+LPS)	0.167 (0.054)	**0.004 (0.018)**
EtOH 1/40 (+LPS)	1.000 (0.874)	**0.020** (0.163)
AM70 1/10 (+LPS)	**< 0.001 (< 0.001)**	**0.004 (< 0.001)**
AM70 1/20 (+LPS)	**0.001 (< 0.001)**	**< 0.001 (0.011)**
AM70 1/40 (+LPS)	**0.002 (0.027)**	**0.044** (0.065)
AM0 1/10 (+LPS)	**0.007 (0.012)**	0.979 (0.741)
AM0 1/20 (+LPS)	0.951 **(< 0.001)**	0.588 **(< 0.001)**
AM0 1/40 (+LPS)	0.277 **(0.002)**	0.875 (0.070)

†= Asymp. sig. Mann Whitney U-test (n=3); EtOH: Ethanol, AM70: *A. millefolium* extract in 70% ethanol; AM0: aqueous *A. millefolium* extract; LPS: lipopolysaccharide; HEK: modified human embryonic kidney cells; HT-29: Human colon tumorigenic cell line clone #16 cells.

A)

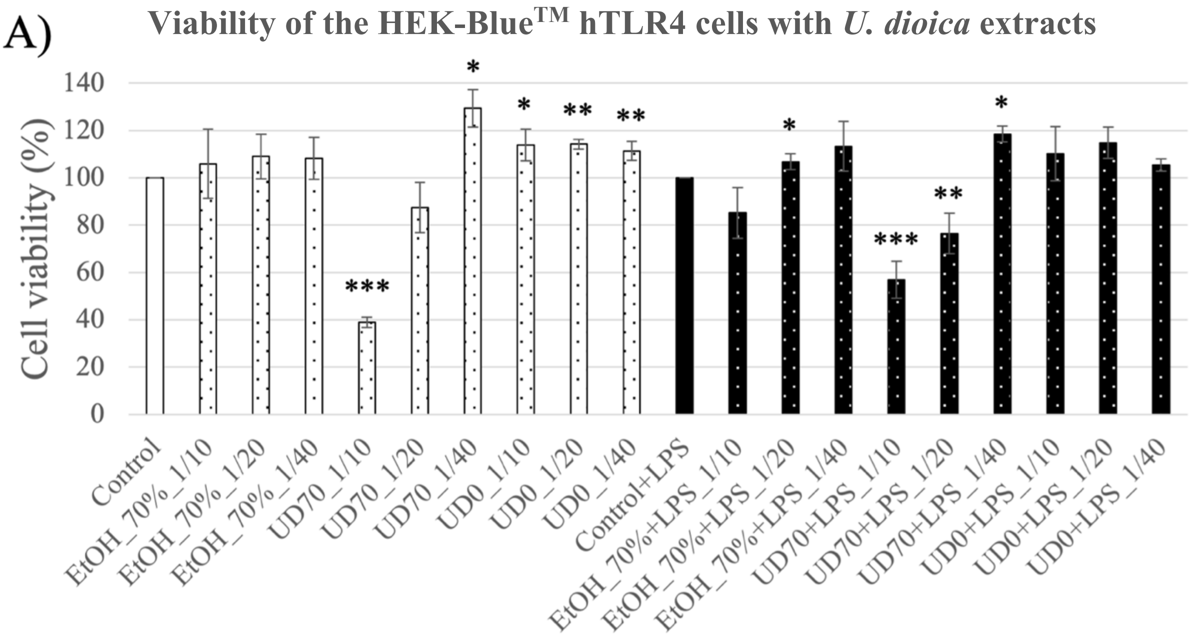

B)

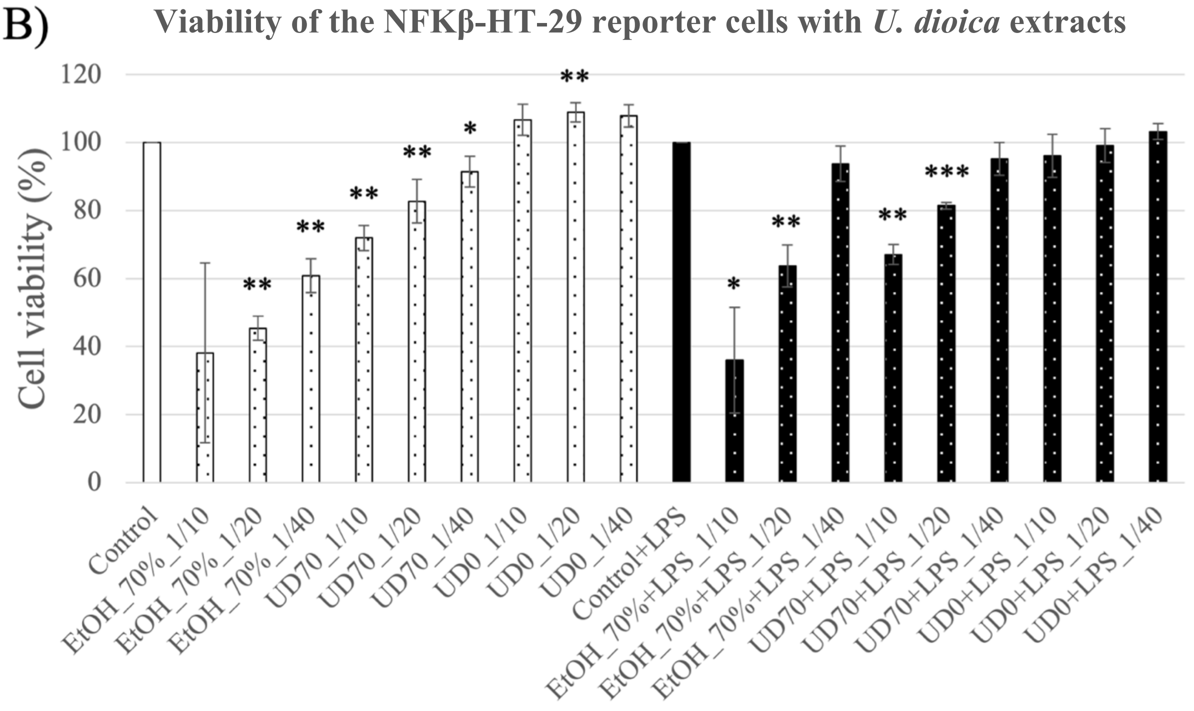

ESM4 Fig. S1 *Viability of HEK-Blue™ hTLR4 cells (A) and NFKβ-HT-29 reporter cell line (B) with different dilutions of U. dioica extracts. Controls were set as 100% and the ethanol and extracts were compared to respective control (with or without LPS), the asterisks show significant differences: * $p < 0.05$, ** $p < 0.01$, *** $p < 0.001$. The control with LPS was 95 % of the Control in HEK-Blue™ hTLR4 and 105 % in NFKβ-HT-29 cells. Data represent means with standard deviation (n = 3). EtOH: Ethanol; UD: U. dioica in 70 % Ethanol or 0 % as aqueous; LPS: Lipopolysaccharide.*

ESM4 Table S2 *The p values of cell controls compared with the ethanol controls and U. dioica extracts for of HEK-Blue™ hTLR4 and NFKβ-HT-29 reporter cell viabilities (ESM4 Fig S2). The bolded values show statistical significance at the level of 0.05 with a T-Test. Values of lipopolysaccharide induction are inside brackets.*

	p values	
Extract	Control *HEK-Blue™ hTLR4* (with LPS)	Control *NFKβ-HT-29* (with LPS)
EtOH 1/10 (+LPS)	0.555 (0.073)	0.056 **(0.019)**
EtOH 1/20 (+LPS)	0.171 **(0.024)**	**0.001 (0.009)**
EtOH 1/40 (+LPS)	0.179 (0.159)	**0.005** (0.105)
UD70 1/10 (+LPS)	**< 0.001 (< 0.001)**	**0.006 (0.003)**
UD70 1/20 (+LPS)	0.178 **(0.009)**	**0.009 (< 0.001)**
UD70 1/40 (+LPS)	**0.024 (0.011)**	**0.030** (0.227)
UD0 1/10 (+LPS)	**0.024** (0.262)	0.129 (0.400)
UD0 1/20 (+LPS)	**0.007** (0.061)	**0.006** (0.757)
UD0 1/40 (+LPS)	**0.007** (0.071)	0.055 (0.137)

EtOH: Ethanol, UD70: *U. dioica* extract in 70% ethanol; UD0: aqueous *U. dioica* extract; LPS: lipopolysaccharide; HEK: modified human embryonic kidney cells; HT-29: Human colon tumorigenic cell line clone #16 cells.
